# Crystal Violet Staining Alone Is Not Adequate to Assess Synergism or Antagonism in Multi-Species Biofilms of Bacteria Associated With Bacterial Vaginosis

**DOI:** 10.3389/fcimb.2021.795797

**Published:** 2022-01-05

**Authors:** Joana Castro, Ângela Lima, Lúcia G. V. Sousa, Aliona S. Rosca, Christina A. Muzny, Nuno Cerca

**Affiliations:** ^1^ Centre of Biological Engineering (CEB), Laboratory of Research in Biofilms Rosário Oliveira (LIBRO), University of Minho, Braga, Portugal; ^2^ Division of Infectious Diseases, University of Alabama at Birmingham, Birmingham, AL, United States

**Keywords:** bacterial vaginosis, anaerobic bacteria, biofilm quantification, microtiter plates, crystal violet staining

## Abstract

Bacterial Vaginosis (BV) involves the presence of a multi-species biofilm adhered to vaginal epithelial cells, but its in-depth study has been limited due to the complexity of the bacterial community, which makes the design of *in vitro* models challenging. Perhaps the most common experimental technique to quantify biofilms is the crystal violet (CV) staining method. Despite its widespread utilization, the CV method is not without flaws. While biofilm CV quantification within the same strain in different conditions is normally accepted, assessing multi-species biofilms formation by CV staining might provide significant bias. For BV research, determining possible synergism or antagonism between species is a fundamental step for assessing the roles of individual species in BV development. Herein, we provide our perspective on how CV fails to properly quantify an *in vitro* triple-species biofilm composed of *Gardnerella vaginalis*, *Fannyhessea (Atopobium) vaginae*, and *Prevotella bivia*, three common BV-associated bacteria thought to play key roles in incident BV pathogenesis. We compared the CV method with total colony forming units (CFU) and fluorescence microscopy cell count methods. Not surprisingly, when comparing single-species biofilms, the relationship between biofilm biomass, total number of cells, and total cultivable cells was very different between each tested method, and also varied with the time of incubation. Thus, despite its wide utilization for single-species biofilm quantification, the CV method should not be considered for accurate quantification of multi-species biofilms in BV pathogenesis research.

## Introduction

Biofilms are widely present in the environment (Hall-Stoodley et al., 2004), industry settings (Galié et al., 2018), and are causative agents of human infections (Vestby et al., 2020). A biofilm can be defined as a three-dimensional microbial community that grows on an abiotic or biotic surface, and is surrounded by an exopolymer matrix composed of bacterial- and environmental-derived molecules (Flemming et al., 2016). This matrix composition can vary with time and is dependent on the bacterial species present in the biofilm, as well as environmental conditions (Flemming and Wingender, 2010). The biofilm matrix is an important protective barrier against external stimuli, including antimicrobial agents (Sharma et al., 2019). However, the matrix is not solely responsible for antimicrobial tolerance, with biofilm heterogeneity (Hall and Mah, 2017) and reduced metabolism (Crabbé et al., 2019) other key factors.

It is widely acknowledged that a polymicrobial biofilm is the hallmark of bacterial vaginosis (BV) (Verstraelen and Swidsinski, 2019), the leading vaginal infection in women of childbearing age (Redelinghuys et al., 2020). BV can lead to serious obstetric and gynecological complications. Furthermore, Women with BV are at increased risk for acquisition of HIV (Atashili et al., 2008) and other STIs, including *Chlamydia trachomatis* and *Trichomonas vaginalis* (Abbai et al., 2016), *Neisseria gonorrhoeae* (Brotman et al., 2010), *Mycoplasma genitalium* (Lokken et al., 2017), human papilloma virus (HPV) (Brusselaers et al., 2019), and herpes simplex virus type-2 (HSV-2) (Abbai et al., 2018). Despite its importance, BV etiology remains undetermined and a matter of controversy (Chen et al., 2021) and the study of polymicrobial biofilms associated with BV is still in its infancy. It has been hypothesized that virulent strains of *Gardnerella* spp. initiate the formation of the biofilm on vaginal epithelial cells and become a scaffolding to which other BV-associated bacteria (BVAB) can attach thereafter (Machado and Cerca, 2015). In fact, an earlier study showed that *Gardnerella* spp. produce amino acids through their metabolism, which can be used by *Prevotella bivia* as its nutrient source which results in the production of ammonia, which in turn is used by *Gardnerella* spp. (Pybus and Onderdonk, 1997). It has also been recently hypothesized that, as a result of these initial bacterial interactions, the vaginal epithelium might be damaged by losing the protective mucous layer, being more favorable for the adherence of other BV-associated bacteria (Muzny et al., 2019). To validate this hypothesis, the experimental determination of synergistic or antagonistic interactions within multi-species BV biofilms is fundamental.

Due to the pivotal role of *Gardnerella* spp. in BV biofilms (Swidsinski et al., 2005), we have sought to quantify *in vitro* BV-associated biofilms, by using a model that first allows *Gardnerella* spp. to establish a biofilm, followed by the addition of other BVAB to the pre-formed *Gardnerella vaginalis* biofilm. Until recently (Rosca et al., 2020) we have not assessed single-species biofilm formation by other BVAB beyond *G. vaginalis*, as this is not a naturally occurring phenomenon. In an early dual-species study using this model, we have identified possible synergism and antagonism between several BVAB (Castro and Cerca, 2015). However, this assessment was only performed using the crystal violet (CV) staining method that, despite being the most widely used technique to quantify biofilms, is not without its flaws (Azeredo et al., 2017). Moving forward to studying triple-species biofilms, we observed that CV staining failed to predict important interactions occurring within these consortia (Castro et al., 2021). Since there is a lack of critical studies comparing the different methodological approaches to quantifying multi-species biofilms (Magana et al., 2018), we aimed to provide a perspective on the lack of feasibility of the CV method to properly assess possible synergism or antagonism between individual BV-associated bacteria growing as triple-species biofilms. For this purpose, we quantified single-species biofilms formed by three BVAB thought to play significant roles in the pathogenesis of incident BV (Muzny et al., 2019), namely *G. vaginalis*, *Fannyhessea vaginae* (previously known as *Atopobium vaginae*) (Nouioui et al., 2018), and *P. bivia.*

## Materials and Methods

### Bacterial Strains and Culture Conditions

*G. vaginalis* strain ATCC 14018^T^, *F. vaginae* strain ATCC BAA-55^T^, and *P. bivia* strain ATCC 29303^T^ were used in this study. Each inoculum was grown in New York City III broth (NYC III) [1.5% (wt/vol) Bacto proteose peptone no. 3 (BD, NJ, USA); 0.5% (wt/vol) glucose (Thermo Fisher Scientific, KS, USA); 0.24% (wt/vol) HEPES (VWR, NV, USA); 0.5% (wt/vol) NaCl (VWR); 0.38% (wt/vol) Yeast extract (Liofilchem, Roseto degli Abruzz, Italy)], supplemented with 10% (vol/vol) inactivated horse serum (Biowest, Nuaillé, France) for 24 h at 37°C under anaerobic conditions (AnaeroGen Atmosphere Generation system, Oxoid, United Kingdom), as optimized before (Rosca et al., 2020).

### Single- and Multi-Species BV Biofilm Formation Model

Single-species biofilms were initiated by inoculating a 10^7^ CFU.mL^-1^ bacterial suspension of each tested bacterial species into 24-well tissue culture plates (Orange Scientific, Braine L’Alleud, Belgium) and incubating the plates for 24 or 48 h at 37°C under anaerobic conditions. Of note, we first adjusted the bacterial concentration of the bacterial suspension to 9 × 10^7^ CFU.mL^-1^ due to the limit of detection of the microplate reader, and then diluted it to 1 × 10^7^ CFU.mL^-1^. At 620 nm, 9 × 10^7^ CFU.mL^-1^ of *G. vaginalis* corresponds to an optical density (OD) of 0.15; *F. vaginae* an OD of 0.11, and *P. bivia* an OD of 0.16 (Castro et al., 2021). Multi-species biofilms were also initiated by inoculating a 10^7^ CFU.mL^-1^ bacterial suspension of *G. vaginalis* into 24-well tissue culture plates and incubating the plates for 24 h at 37°C under anaerobic conditions. After 24 h, planktonic cells were removed, and 10^7^ CFU.mL^-1^ of *F. vaginae* and *P. bivia* were inoculated in the pre-formed *G. vaginalis* biofilms, followed by another 24 h of incubation (**Supplementary Figure 1**). As a control, single-species biofilms of *G. vaginalis* were grown for 24 and 48 h, in which fresh medium was added to the respective wells after the first 24 h of biofilm formation (for the 48-h control). These assays were repeated at least three times on separate days.

### Biofilm Biomass Quantification by the Crystal Violet (CV) Method

To quantify the biomass of single- and multi-species biofilms, we used the CV method (Peeters et al., 2008). In brief, after the fixation step with 100% (vol/vol) methanol (Thermo Fisher Scientific) for 20 min, biofilms were stained with CV solution at 1% (vol/vol) (Merck, Darmstadt, Germany) for 20 min. Each well was washed twice with PBS, and bound CV was released with 33% (vol/vol) acetic acid (Thermo Fisher Scientific). To estimate total biofilm biomass, the OD of the resulting solution was measured at 595 nm. Biofilm assays were repeated at least three times on separate days, with four technical replicates assessed each time.

### Quantification of Total Number of Cells in the Biofilm Using Acridine Orange Through Epifluorescence Microscopy

Prior to the quantification of total biofilm cells, several optimizations were performed. First, we prepared fresh suspensions of each bacterial species from Columbia Blood Agar (CBA) plates and then adjusted the bacterial concentration to 10^8^ CFU.mL^-1^. We subsequently performed several dilutions in PBS 1×, aiming to determine the number of fields needed to obtain linearity among the different dilutions (**Supplementary Figure 2**). A minimum of 13 images per sample resulted in a very high correlation between bacterial counts and bacterial concentration. After this first optimization, we quantified the total number of cells from the single- and multi-species biofilms. In brief, the biofilms were carefully washed with 0.9% (wt/vol) NaCl, and 1 mL of PBS 1× was added to each well. The biofilms were then scrapped, and a pool of the different wells was obtained. Afterward, 30 µL of each bacterial suspension dilution was spread on epoxy-coated microscope glass slides (Thermo Fisher Scientific), and the slides dried at 60°C. Next, cells were fixed at room temperature with 100% (vol/vol) methanol for 20 min, followed by 4% (wt/vol) paraformaldehyde (Thermo Fisher Scientific) for 10 min, and 50% (vol/vol) ethanol (Thermo Fisher Scientific) for 15 min. After the fixation step, the samples were covered with 20 µL of acridine orange (0.01 mg.mL^-1^) for 5 min. The excess of acridine orange was removed and the slides were air-dried in the dark at room temperature. Microscope visualization was performed using filters capable of detecting acridine orange (BP 470-490, FT500, LP 516). The number of bacterial cells was manually counted, at the appropriate dilution (<100 bacteria per field). These assays were repeated three times on separate days.

### Enumeration of Total Culturable Bacteria in the Biofilm Using the CFU Counting Method

Regarding the culture plate counting method, serial dilutions ranging from 10^-1^ to 10^-6^ were performed on the resuspended biofilm in 0.9% (wt/vol) NaCl. After homogenization, 10 µL of each dilution was spread onto CBA plates. The plates were incubated at 37°C under anaerobic conditions for 72 h. This process was carried out with two replicates in at least three independent assays. More details are explained in the Supplementary Materials and Methods.

### Discrimination of Bacterial Populations in Multi-Species Biofilms by PNA-FISH

The bacterial population within the 48 h multi-species biofilms was discriminated using the peptide nucleic acid fluorescence *in situ* hybridization (PNA-FISH) method, as previously described (Castro et al., 2021). Briefly, after fixing the biofilm suspension, a PNA probe specific for *G. vaginalis* (Gard162) and for *F. vaginae* (AtoITM1) were added to each well of epoxy-coated microscope glass slides (Thermo Fisher Scientific). An additional staining step was done at the end of the hybridization procedure, covering each glass slide with DAPI (2.5 μg.mL^-1^). Microscopic visualization was performed using filters capable of detecting the PNA Gard162 probe (BP 530-550, FT 570, LP 591 sensitive to the Alexa Fluor 594 molecule attached to the Gard162 probe), the PNA AtoITM1 probe (BP 470-490, FT500, LP 516 sensitive to the Alexa Fluor 488 molecule attached to the AtoITM1 probe), and DAPI (BP 365–370, FT 400, LP 42). The number of bacteria was counted using *ImageJ Software* (Rasband, 1997). These assays were repeated three times on separate days.

### Statistic Analysis

The data were analyzed using GraphPad Prism version 7 (La Jolla,CA, USA) by unpaired t-test, or non-parametric Wilcoxon matched-pairs signed-rank test. A *P* < 0.05 were considered statistically significant. Data are presented as mean (of all independent assays) ± standard deviation (s.d.).

## Results and Discussion

**Figure 1:**
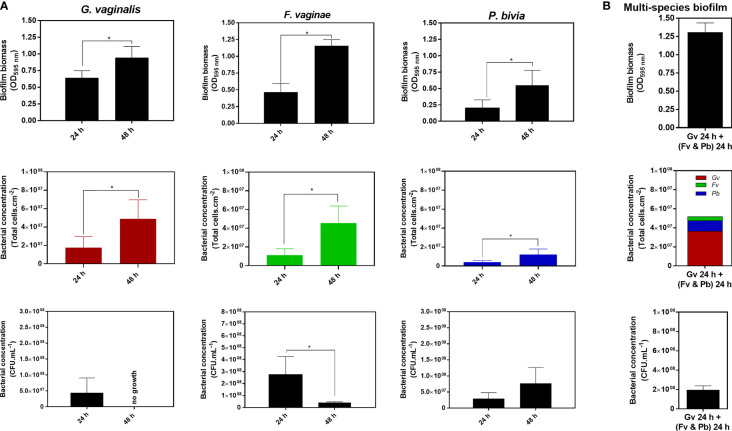
Quantification of 24 h and 48 h single-species biofilms of *G. vaginalis*, *F. vaginae* and *P. bivia* **(A)** or a multi-species biofilm composed of all three species **(B)** using the crystal violet method, total cell counts by epifluorescence microscopy and the colony-forming units (CFU) method. The colors selected for the epifluorescence microscopy data reflect the fluorophore detection spectra. Each data point represents the average ± s.d. of three experiments. *Values are significantly different between 24 h and 48 h of biofilm formation without changing the growth medium (batch system) (unpaired t-test, *P* < 0.05).

To better understand how different BVAB are affected by standard biofilm quantification, we first characterized 24 and 48 h single-species biofilms to assess how each technique reflects biofilm growth. The total biofilm biomass was determined by the CV method, while cell culturability was detected anaerobically in the appropriate medium and total cells were quantified by epifluorescence microscopy. For all three tested species, we observed that the total biofilm biomass and bacterial concentration obtained by epifluorescence microscopy significantly increased after 48 h of biofilm formation in batch conditions, compared to a 24 h-biofilm (p<0.05) (**Figure 1A**). However, the same was not true for bacterial culturability, wherein only *P. bivia* was able to increase its bacterial concentration from 24 to 48 h biofilms. In contrast, *F. vaginae* significantly decreased its bacterial culturability after 48 h of biofilm formation, while no CFU was able to grow from 48 h-*G. vaginalis* biofilms in the tested conditions.

Since it was previously shown that *G. vaginalis* lost 1 log cell culturability when manipulated (after anaerobic growth) in a regular biosafety cabinet (Turovskiy et al., 2012), we verified if this significant loss of culturability could be a result of bacterial manipulation in the presence of atmospheric oxygen. Two approaches were tested (controlled delay after biofilm scrapping and after CFU plating – see Supplementary Methods and **Supplementary Figure 3**). As shown in **Supplementary Figure 4**, the reduction of bacterial culturability was more affected by the delay after plating the suspension onto CBA plates. Still, by performing all manipulations under 30 min, we were able to reduce the loss of viability under 20%, which does not explain the observed significantly higher loss of culturability. As recently pointed out for bacterial species in the gut microbiota, a possible reason that a greater proportion of the bacterial community was not cultured when the fresh sample was exposed to O_2_ might be the fact that oxygen-sensitive cells were in the viable but not culturable (VBNC) state, or either injured or dead (Bellali et al., 2019). While similar observations have been reported elsewhere (Li et al., 2014; Lv et al., 2020), to our knowledge, this has not been determined in *G. vaginalis* biofilms. As such, we performed another experiment, wherein we used the LIVE/DEAD^®^ *Bac*Light™ Bacterial Viability Kit. It is important to highlight that this staining system has some limitations, as pointed by Netuschil and colleagues, mainly when used in multi-species biofilms (Netuschil et al., 2014). However, in this study, we only used the LIVE/DEAD kit for the examination of single-species biofilms, in which carrying out adequate controls allows obtaining reliable interpretations (Robertson et al., 2019). By using this kit, we were able to observe viable cells within the 48 h *G. vaginalis* biofilms, although the majority of cells had damaged cell walls (**Supplementary Figure 5**). The presence of this state has been associated with longer periods of biofilm formation, nutritional resource limits, and deposits of metabolic waste (Ayrapetyan et al., 2018; Carvalhais et al., 2018). Interestingly, we could prevent VBNC in 48 h *G. vaginalis* biofilms by replacing the growth media after 24 h (**Supplementary Figure 5**).

Interestingly, after comparing the quantification of 24 or 48 h biofilms by the three different techniques, we observed that each species had significantly different quantification yields, depending on the technique used. Clearly, the total biomass (cells plus matrix) produced by the different species varied among species and with the time of incubation (**Table 1A**). Such a fact is not surprising, given that these techniques measure different features of the biofilm (Stiefel et al., 2016). To better highlight the differences found in the quantification of each single-species biofilm, we calculated the ratio of biofilm total biomass formation by 1×10^7^ total cell.cm^-2^ or by 1×10^7^ CFU.mL^-1^. Curiously, our findings indicated that under our tested conditions, *G. vaginalis* produced the lowest biofilm biomass by each 1×10^7^ total cell.cm^-2^, which became more pronounced at 48 h. In contrast, *P. bivia* produced more biofilm biomass per bacteria (**Table 1B**). While the CV method for the quantification of the biofilm biomass is widespread (Azeredo et al., 2017), it has been suggested that direct comparison of total biofilm biomass between species might not be feasible, as different species may have distinct biofilm matrices (Haney et al., 2018). This is supported by the results of this study. To make comparisons even more challenging, the ratio of biofilm biomass produced at different incubations times might not be constant, at least for *G. vaginalis* and *F. vaginae* in our tested conditions.

**Table 1:** Different ratios resulted from quantification using the three different methodologies.

**PANEL A** | Ratios obtained from data of the quantification of 48 h and 24 h single-species biofilms by three different methodologies			
	***G. vaginalis***	***F. vaginae***	***P. bivia***
**Biofilm biomass (48 h/24 h)**	1.47	2.48	2.81
**Total cell counts by epifluorescence microscopy (48 h/24 h)**	2.77	4.03	2.95
**CFU counting (48 h/24 h)**	0.0	0.15	2.59
**PANEL B** | Ratios obtained from data of the quantification of total cells or CFUs (both expressed in 1×10^7^) in relation to biofilm biomass for each time point assessed for single-species biofilms			
	***G. vaginalis***	***F. vaginae***	***P. bivia***
**Biofilm biomass/Total cell counts by epifluorescence microscopy (24 h)**	0.36	0.41	0.47
**Biofilm biomass/Total cell counts by epifluorescence microscopy (48 h)**	0.19	0.25	0.45
**Biofilm biomass/CFU counting (24 h)**	0.15	0.02	0.07
**Biofilm biomass/CFU counting (48 h)**	n.d.*	0.28	0.07
**PANEL C** | Ratios obtained from data of the quantification of total cells or CFUs (both expressed in 1×10^7^) in relation to biofilm biomass for multi-species biofilms			
	**Multi-species biofilm**		
**Biofilm biomass/Total cell counts by epifluorescence microscopy**	0.25		
**Biofilm biomass/CFU counting**	0.07		

*n.d., not determined – Since the value of cfu.mL^-1^ for the 48 h G. vaginalis biofilm was zero, it was not possible to determine the ratio.

The results of this study raise the question of how the CV staining method of a multi-species biofilm could in fact reflect its bacterial composition. As shown in **Table 1C**, multi-species biofilms had very distinct CV/total cells, or CV/CFU ratios, further suggesting that simply quantifying a multi-species biofilm by the CV method will not provide a reliable quantification of the biofilm. While both the absolute CV (**Figure 1B**) staining and the CV/total cells ratio were similar to the 48 h *F. vaginae* biofilm, it is very unlikely that this multi-species biofilm would be solely composed by *F. vaginae*. Furthermore, the CV/total cells ratio did not match the *F. vaginae* profile. With this in mind, we analyzed the bacterial composition in the multi-species by PNA-FISH differentiation, using specific probes for *G. vaginalis* and *F. vaginae* and DAPI, counterstaining to quantify total cells (Castro et al., 2021). Under our tested conditions, *G. vaginalis* represented 70.3 ± 1.2% of the multi-species biofilm, followed by *P. bivia* (21.4 ± 1.0%) and *F. vaginae* (8.3 ± 0.9%).

## Conclusions and Perspectives

CV staining quantification has proven extremely useful as a cellular estimate for biofilm formation, mainly because both Gram-positive and Gram-negative bacterial cells are able to take up the CV. The dye will freely pass from the cell during the decolorization process, allowing for the quantification of CV *via* spectroscopy (Peeters et al., 2008; Magana et al., 2018). However, it has been noted that in polymicrobial consortia, accurate biofilm quantification becomes more complex (Røder et al., 2016).

As shown here, our three key BVAB produced different biofilms with different profiles (i.e. cells and matrix), which varied with time (with the exception of *P. bivia*). The relationship between total biofilm biomass/total cells is unique to each tested species, in specific environmental conditions, and as such, a direct comparison between single- and multi-species biofilms using the CV method alone is unlikely to be without bias. If we could assume that, for a specific period of incubation, each individual species could maintain the same biofilm production profile, when growing alone or in consortia, it might be possible to interpolate the measured data to be adjusted by the relative contribution of the species in the multi-species biofilm. However, when growing in consortia, the biofilm matrix components produced by each species might be affected, since the matrix composition is highly dependent on environmental conditions (Karygianni et al., 2020). Thus, we proposed that when comparing single to multiple-species biofilms, an increased or decreased CV staining should not be taken as an accurate measure of bacterial synergism or antagonism, as we have mistakenly done before (Castro and Cerca, 2015). A lower total biomass might in fact reflect an increase in cell concentration. On the other hand, an increase in total biomass might provide an advantage to the cells within the biofilm, by providing better protection against antibiotics (Sharma et al., 2019), even if the total bacterial load is reduced. Due to this complexity, we argue that to properly analyze a BV-associated, or in that matter any other multi-species biofilms, a multiple-technical approach should be used when quantifying these consortia, in order to circumvent the caveats of individual techniques alone. This multiple technical approach will provide a more compressive picture of the biofilm consortia associated with BV, and will contribute in furthering BV pathogenesis research.

## Data Availability Statement

The raw data supporting the conclusions of this article will be made available by the authors, without undue reservation.

## Author Contributions

NC and CM designed the experiments. JC, ÂL, LS, and AR performed the experiments. JC and NC drafted the manuscript. All authors critically reviewed and approved the final manuscript.

## Funding

This research was partially funded by the Portuguese Foundation for Science and Technology (FCT) by the research project (PTDC/BIA-MIC/28271/2017), under the scope of COMPETE 2020 (POCI-01-0145-FEDER-028271), by the strategic funding of unit (UIDB/04469/2020). It was also partially funded by the National Institute of Allergy and Infectious Diseases (R01AI146065-01A1). The funders had no role in study design, data collection and analysis, decision to publish, or preparation of the manuscript.

## Conflict of Interest

CM has received research grant support from Lupin Pharmaceuticals, Gilead, and Abbott Molecular, is a consultant for Lupin Pharmaceuticals, PhagoMed, and BioFire Diagnostics, and has received honoraria from Elsevier, Abbott Molecular, Cepheid, Becton Dickinson, Roche Diagnostics, and Lupin.

The remaining authors declare that the research was conducted in the absence of any commercial or financial relationships that could be construed as a potential conflict of interest.

## Publisher’s Note

All claims expressed in this article are solely those of the authors and do not necessarily represent those of their affiliated organizations, or those of the publisher, the editors and the reviewers. Any product that may be evaluated in this article, or claim that may be made by its manufacturer, is not guaranteed or endorsed by the publisher.
