# Supplementary material for: Crystal Violet Staining Alone Is Not Adequate to Assess Synergism or Antagonism in Multi-Species Biofilms of Bacteria Associated With Bacterial Vaginosis
Source: Front Cell Infect Microbiol. 2022 Jan 5;11:795797. doi: 10.3389/fcimb.2021.795797 (PMC8766793; doi:10.3389/fcimb.2021.795797)
Supplement: Supplementary file 1 [file DataSheet_1.pdf]

Supplementary Material

Crystal violet staining alone is not adequate to assess synergism or antagonism in multi-species biofilms of bacteria associated with bacterial vaginosis

Joana Castro[1], Ângela Lima[1], Lúcia G. V. Sousa[1], Aliona S. Rosca[1], Christina A. Muzny[2], Nuno Cerca[1,*]

[1] Centre of Biological Engineering (CEB), Laboratory of Research in Biofilms Rosário Oliveira (LIBRO), University of Minho, Campus de Gualtar, 4710-057 Braga, Portugal

[2] Division of Infectious Diseases, University of Alabama at Birmingham, Birmingham, AL 35294, USA

SUPPLEMENTARY MATERIALS AND METHODS

Enumeration of total culturable bacteria in the biofilm using the CFU counting method

After the time of biofilm formation (24 h or 48 h), biofilms were carefully washed with 0.9% (wt/vol) NaCl, and 1 mL of NYC III was added into each well. The biofilms were then scrapped, and a pool of two different wells was obtained for each condition. Then, for each condition, serial dilutions ranging from 10^{-1} to 10^{-6} were performed on the biofilm pool suspensions diluted in 0.9% (wt/vol) NaCl. After homogenization, 10 µL of each dilution was spread onto Columbia Base Agar supplemented with 5 % (v/v) horse defibrinated blood (CBA) plates and then incubated at 37 °C under anaerobic conditions for 72 h. After this period, the colonies were counted in the appropriate dilution and the bacterial concentration was calculated accordingly. Of note, we only assessed one condition each time (time of procedure was approx. 15 min) to avoid misleading data interpretation due to the effect of atmosphere conditions (O_2 exposition).

Effect of the time and manipulation process on bacterial enumeration of anaerobes from single-species biofilms by the CFU counting method using two different approaches

Single-species biofilms were formed, as described in the main text. After 24 h, the biofilms were carefully washed with 0.9% (wt/vol) NaCl, and 1 mL of NYC III was added into each well. The biofilms were then scrapped, and a pool of four different wells was obtained, as detailed in Supplementary Figure 3. Total culturable cells number was obtained by the plate counting method, in which we tested the effect of manipulation of these anaerobic bacterial species using two different approaches: delay after scrapping the bacterial cells from the biofilm *(i)*; delay after plating the bacterial suspension onto CBA *(ii)*. First, for *G. vaginalis*, we assessed different time points, namely 30 min, 60 min, and 90 min. Afterwards, for *F. vaginae* and *P. bivia*, which are strict anaerobic bacteria, only the first time point was assessed (30 min). In each condition, serial dilutions ranging from 10^{-1} to 10^{-6} were performed on the biofilm pool suspensions diluted in 0.9% (wt/vol) NaCl. After homogenization, 10 µL of each dilution was spread onto CBA plates and then incubated at 37

°C under anaerobic conditions for 72 h. It is important to note that in our optimized protocol, collection of a biofilm specimen was obtained after 15 min of exposure of the cells to normal O_2 concentrations (around 21%), given the fact that all the manipulation was done under a flow laminar chamber. This process was carried out with 3 replicates and was repeated at least three times on separate days.

Analysis of the viability of *G. vaginalis* biofilm cells using batch and fed-batch conditions

LIVE/DEAD® *Bac*Light™ Bacterial Viability Kit (Thermo Fisher Scientific) was used to determine the viability of cells from *G. vaginalis* biofilms. Biofilms were formed in an 8-well chamber slide (Thermo Fisher Scientific™ Nunc™ Lab-Tek™, Bohemia, NY, USA) for a period of 48 h, with and without medium change after the first 24 h of biofilm formation, at 37 ºC under anaerobic conditions. After 48 h of incubation, the biofilms coating the chamber slides were gently washed with phosphate-buffered solution (PBS). A dead control was obtained by covering the biofilms with 400 µL of 100% (vol/vol) methanol (Thermo Fisher Scientific) for 30 min. Then, all the tested biofilms were covered with 100 µL of the Live/Dead staining mix, with SYTO 9 and propidium iodide used each in a concentration of 3 $\mu L.mL^{-1}$. Subsequently, the coverslips were incubated for 15 min in the dark at room temperature. Biofilm image was acquired with an Olympus™ FluoView FV1000 (Olympus) confocal laser scanning microscope, using a 40× objective and a resolution of 640 × 640 pixels. The experiment was repeated two times.

SUPPLEMENTARY FIGURES

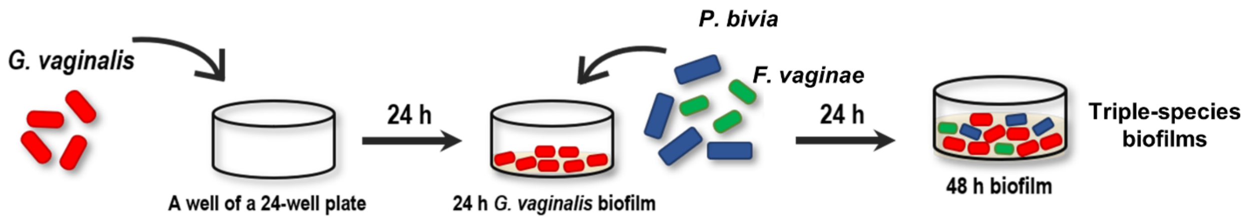

Supplementary Figure 1| Representative multi-species BV-associated biofilm model used in this study. Multi-species biofilms were initiated by inoculating a bacterial suspension of *G. vaginalis* into 24-well tissue culture plates in New York City III (NYC III) medium and by incubating the plates for 24 h, at 37 °C under anaerobic conditions. After 24 h, planktonic cells were removed, and each additional bacterial species, *F. vaginae* and *P. bivia*, were inoculated in the pre-formed *G. vaginalis* biofilms and incubated for another 24 h. Of note that NYC III was preincubated overnight at 37 °C in an anaerobic environment to minimize any stress to the cells (i.e., oxygen, low temperature).

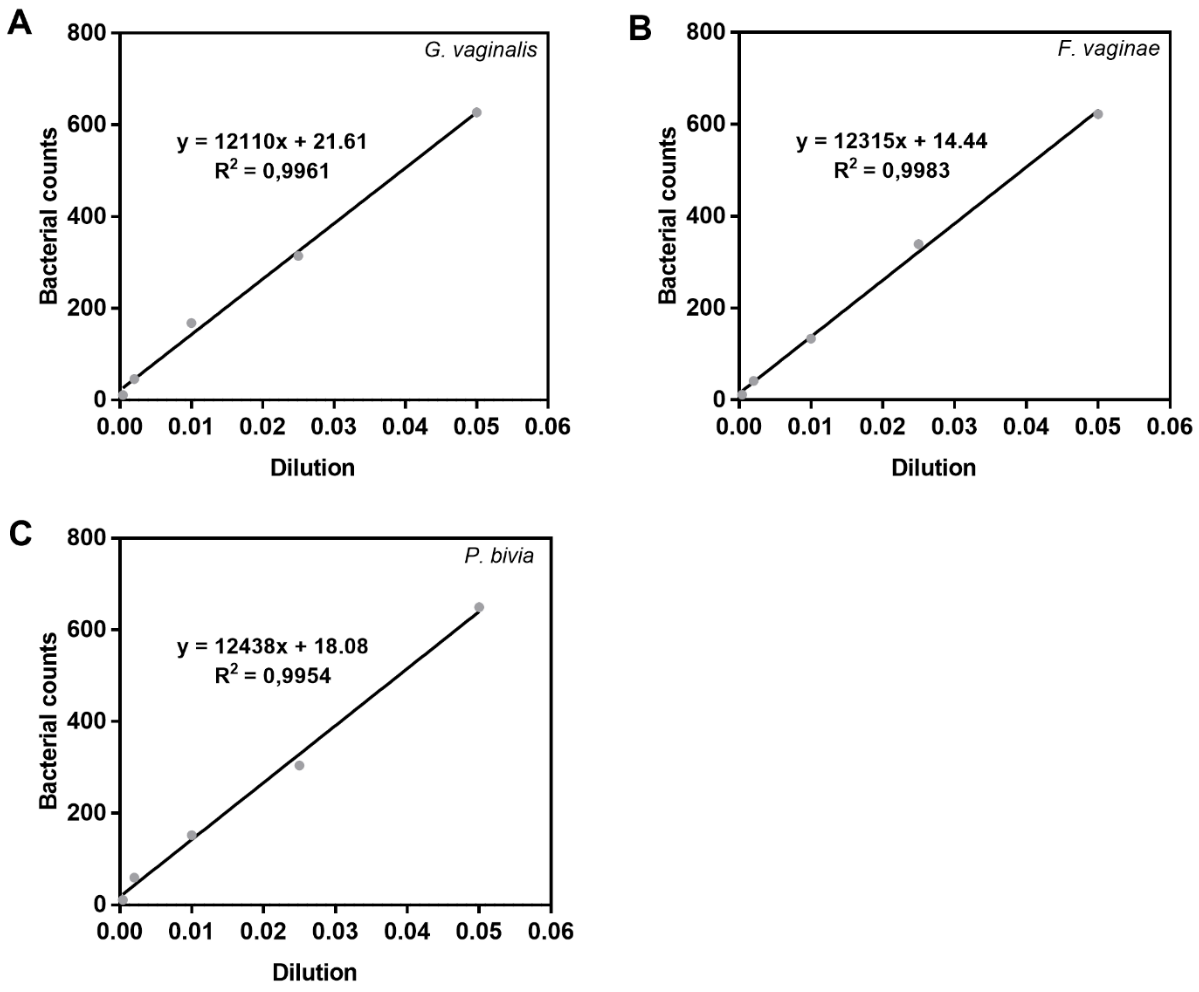

Supplementary Figure 2| Optimization of the quantification of total cells by epifluorescence microscopy using acridine orange. Experiments conducted with *G. vaginalis* **(A)**, *F. vaginae* **(B)** and *P. bivia* **(C)**. Fresh-bacterial suspensions of each bacterial species were prepared from CBA plates, and the bacterial concentration was adjusted to 10^8 CFU.mL^{-1}. We subsequently performed five dilutions (1:20; 1:40; 1:100; 1:500; 1:2500) in PBS 1×, aiming to determine the number of fields needed to obtain linearity among the different dilutions. Thirteen fields were enough to obtain linearity.

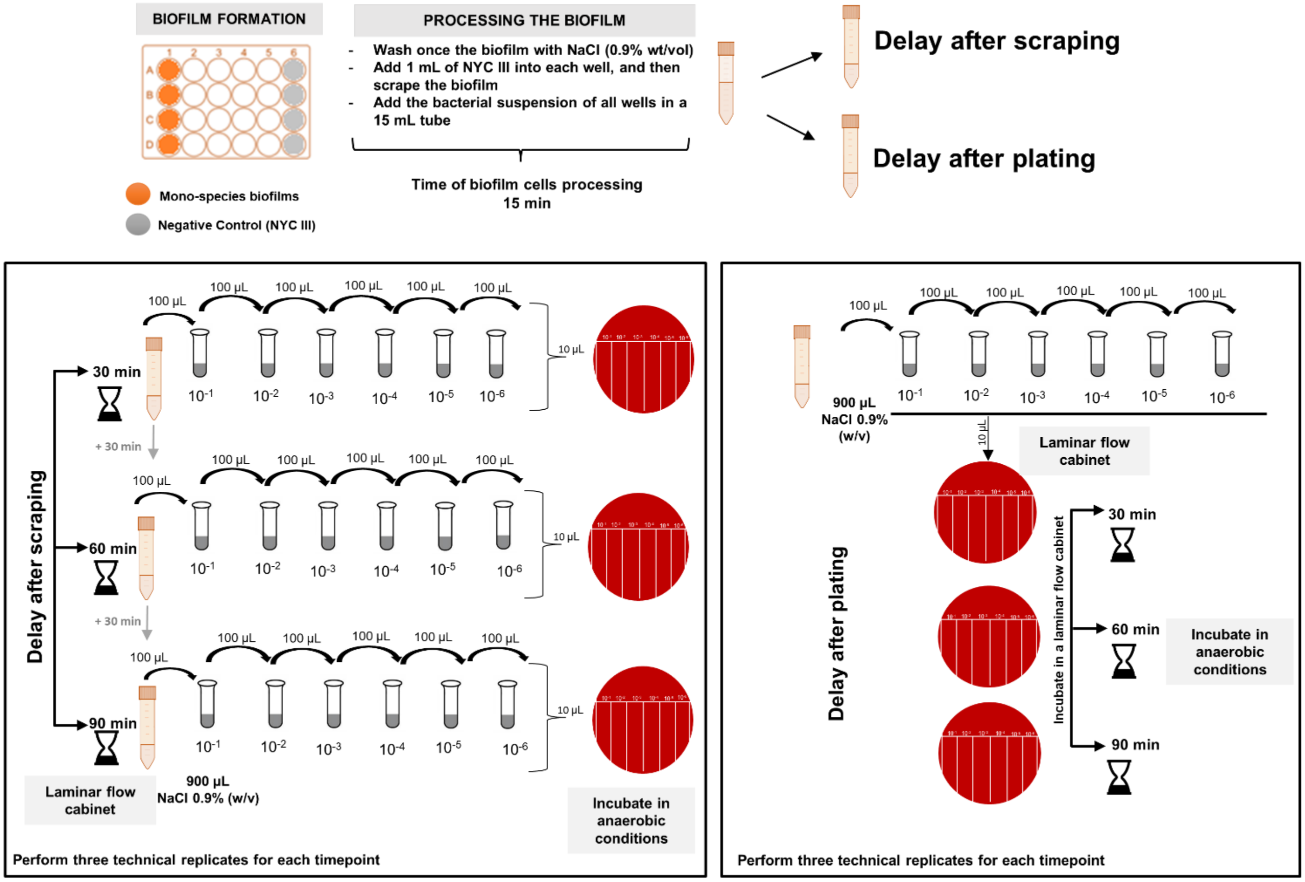

Supplementary Figure 3| Schematic representation of the experimental setup used to assess the influence of oxygen on the culturability of cells obtained from single-species biofilms. The biofilms were scrapped, and a pool of four different wells was obtained. Total culturable cells number was obtained by plate counting method, in which we tested the effect of manipulation of these anaerobic bacterial species using two different approaches: delay after scrapping the bacterial cells from the biofilm *(i)*; delay after plating the bacterial suspension onto Columbia base agar (CBA) plates supplemented with 5% defibrinated horse blood (CBA) *(ii)*. In each condition, serial dilutions ranging from 10^{-1} to 10^{-6} were performed on the biofilm pool suspensions diluted in 0.9 % (wt/vol) NaCl. After homogenization, 10 µL of each dilution was spread onto CBA plates and then incubated at 37ºC under anaerobic conditions for 72 h. It is important to note that in our optimized protocol, biofilm sampling collection was obtained after 15 min of exposure of the cells to normal O_2 concentrations (around 21%), given the fact that the manipulation was done under a flow laminar chamber.

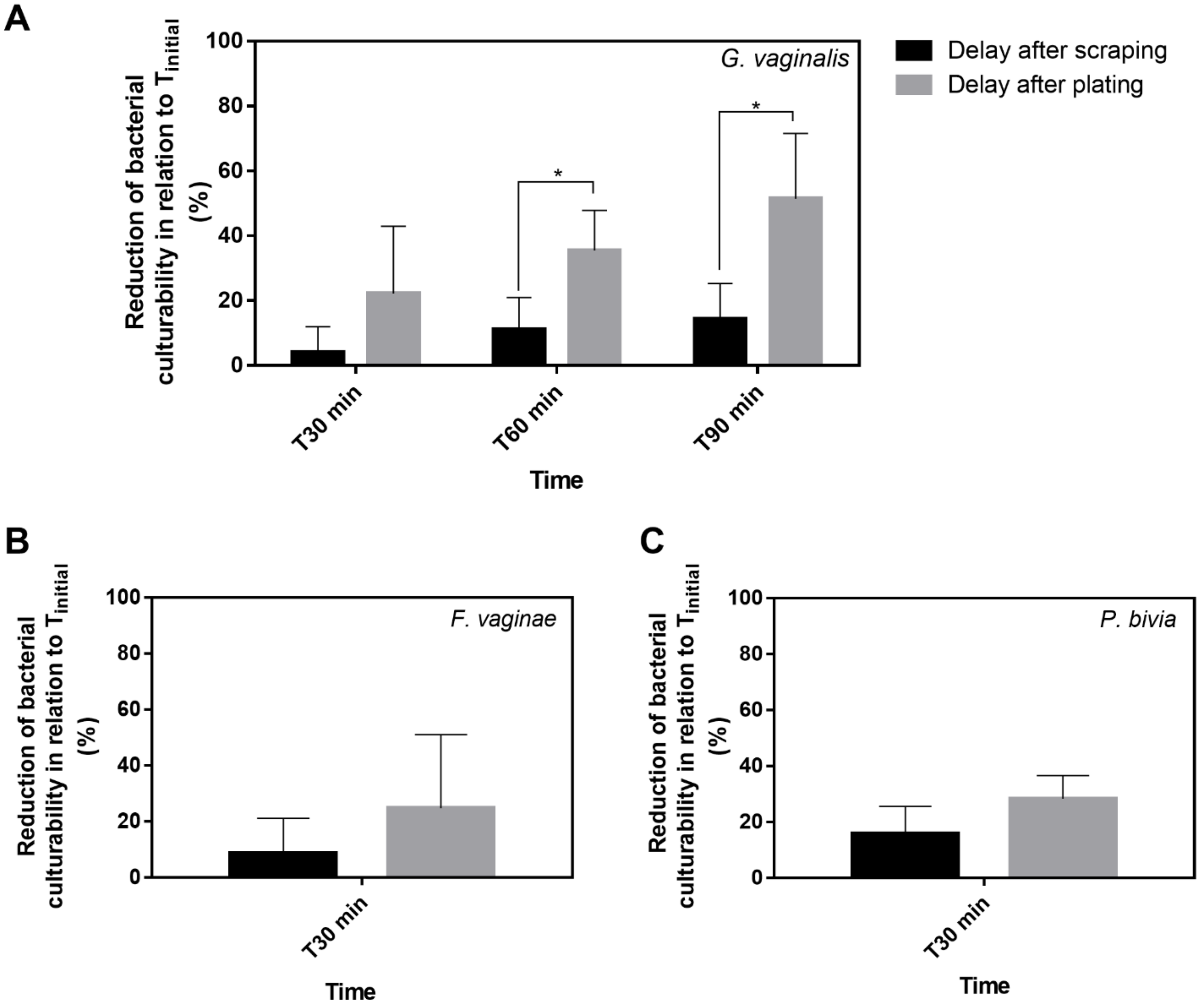

Supplementary Figure 4| Assessment of the reduction of bacterial culturability of BVAB using two different approaches, delay after scraping and delay after plating the bacterial suspension. Experiments were conducted with *G. vaginalis*, in which assessment was performed at three different time points (30, 60, and 90 min) **(A)**. Experiments were also conducted with *F. vaginae* **(B)** and *P. bivia* **(C)**, in which assessment was performed at one-time point (30 min). In each condition, serial dilutions ranging from 10^{-1} to 10^{-6} were performed on the biofilm pool suspensions diluted in 0.9% (wt/vol) NaCl. After homogenization, 10 µL of each dilution was spread onto CBA plates and then incubated at 37 ºC under anaerobic conditions for 72 h. It is important to note that in our optimized protocol, biofilm sampling collection was obtained after 15 min of exposure of the cells to normal O_2 concentrations (around 21%), given the fact that the manipulation was done under a flow laminar chamber. Each data point represents the average ± s.d. of three experiments. *Values are significantly different between the two approaches (Wilcoxon matched-pairs signed-rank test, $P < 0.05$).

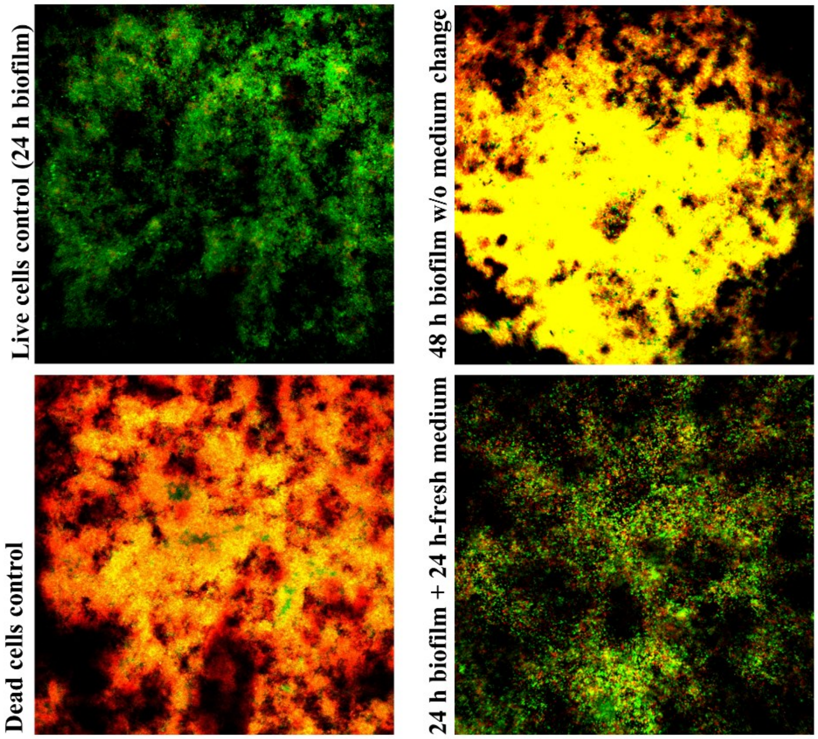

Supplementary Figure 5| Analysis of the viability of *G. vaginalis* biofilm cells using two different biofilm models: with and without growth medium changed. LIVE/DEAD® *Bac*Light™ Bacterial Viability Kit consisting of SYTO 9 and propidium iodide, was used to determine the viability of cells from *G. vaginalis* biofilms. Biofilms were formed in an 8-well chamber slide (Thermo Fisher Scientific™ Nunc™ Lab-Tek™, Bohemia, NY, USA) for a period of 48 h, with and without medium change after the first 24 h of biofilm formation, at 37 ºC under anaerobic conditions. After 48 h of incubation, the biofilms coating the chamber slides were gently washed with 1x PBS. Controls represented by dead and live biofilm cells were used for this experiment. The dead control was obtained by covering the biofilms with 400 μL of 100% (vol/vol) methanol (Thermo Fisher Scientific) for 30 min. Then, all the tested biofilms were covered with 100 μL of the Live/ Dead staining mix, with SYTO 9 and propidium iodide used each in a concentration of 3 $\mu L.mL^{-1}$. Subsequently, the chamber slide was incubated for 15 min in the dark at room temperature. Biofilm images were acquired with an Olympus™ FluoView FV1000 (Olympus) confocal laser scanning microscope, using a 40× objective and a resolution of 640 × 640 pixels. Microscopic visualization was performed using lasers capable of detecting SYTO 9 (Laser 488, excitation wavelength 488 nm, emission wavelength 520 nm, BA505-540) and propidium iodide (Laser 559, excitation wavelength 559 nm, emission wavelength 618 nm, BA575-675). The CLSM images were analyzed using the *FV10-ASW 4.0 Viewer Software* (Olympus).
